# Mathematical Modeling and Finite Element Simulation of the M8514-P2 Composite Piezoelectric Transducer for Energy Harvesting

**DOI:** 10.3390/s25103071

**Published:** 2025-05-13

**Authors:** Demeke Girma Wakshume, Marek Łukasz Płaczek

**Affiliations:** Department of Engineering Processes, Automation and Integrated Manufacturing Systems, Silesian University of Technology, Konarskiego 18a Street, 44-100 Gliwice, Poland; marek.placzek@polsl.pl

**Keywords:** energy harvesting, macro fiber composite, natural frequency, amplitude, mode shape, mechanical vibration

## Abstract

This paper focuses on the mathematical and numerical modeling of a non-classical macro fiber composite (MFC) piezoelectric transducer, MFC-P2, integrated with an aluminum cantilever beam for energy harvesting applications. It seeks to harness the transverse vibration energy in the environment to power small electronic devices, such as wireless sensors, where conventional power sources are inconvenient. The P2-type macro fiber composites (MFC-P2) are specifically designed for transverse energy harvesting applications. They offer high electric source capacitance and improved electric charge generation due to the strain developed perpendicularly to the voltage produced. The system is modeled analytically using Euler–Bernoulli beam theory and piezoelectric constitutive equations, capturing the electromechanical coupling in the d31 mode. Numerical simulations are conducted using COMSOL Multiphysics 6.29 to reduce the complexity of the mathematical model and analyze the effects of material properties, geometric configurations, and excitation conditions. The theoretical model is based on the transverse vibrations of a cantilevered beam using Euler–Bernoulli theory. The natural frequencies and mode shapes for the first four are determined. Depending on these, the resonance frequency, voltage, and power outputs are evaluated across a 12 kΩ resistive load. The results demonstrate that the energy harvester effectively operates near its fundamental resonant frequency of 10.78 Hz, achieving the highest output voltage of approximately 0.1952 V and a maximum power output of 0.0031 mW. The generated power is sufficient to drive ultra-low-power devices, validating the viability of MFC-based cantilever structures for autonomous energy harvesting systems. The application of piezoelectric phenomena and obtaining electrical energy from mechanical vibrations can be powerful solutions in such systems. The application of piezoelectric phenomena to convert mechanical vibrations into electrical energy presents a promising solution for self-powered mechatronic systems, enabling energy autonomy in embedded sensors, as well as being used for structural health monitoring applications.

## 1. Introduction

Energy harvesting captures unused energy available in the environment and then converts it into electrical energy to power a small electronic device [1,2,3]. This energy can serve as a power source for autonomous electronic sensors, especially in remote systems. Nowadays, piezoelectric energy harvesting is a very popular field of mechanical and electrical research because of the small electronic devices and sensors that surround us throughout the modern world. The fundamental mechanisms that are used to convert mechanical vibrations into electrical energy are electromagnetic, electrostatic, and piezoelectric. Classical and non-classical piezoelectric energy harvesters (PEHs) are particularly noteworthy due to their simple structure, high electrical output, compactness, and high-efficiency conversion. This energy harvesting method also offers ease of manufacturing [2,3]. To analyze and design the structure of a piezoelectric transducer element, the main challenge is the interaction between electrical and mechanical coupling. Non-classical macro fiber composite (MFC) piezoelectric transducers have better energy harvesting properties than classical piezoelectric ceramic materials (PZT), including the method of coupling to vibrating structures, sensitivity, and cost. Non-classical composites based on MFC and PVDF films have the property of being easily glued to structures with leading low-profile actuators, energy harvesters, and sensors; they are known for their high performance, flexibility, and reliability in a cost-effective design. The MFC was originally invented by NASA in 1999. The MFC began commercial distribution in 2002 through the Smart Material Corporation, the licensed manufacturer and global distributor. Since its introduction, the MFC has undergone continuous improvement and customization to meet evolving application requirements and specific customer needs. The development of macro fiber composites (MFC) has addressed several limitations associated with traditional monolithic piezoelectric ceramic components, particularly mechanical flexibility, durability, integration capabilities, brittleness, a lack of reliability, limited conformability, and PZT not being ideal for dynamic applications [3,4,5]. Recent advances in ultra-low-power electronic components have created a new class of electronic devices: battery-free, autonomous systems that operate solely by harvesting mechanical vibrations through piezoelectric components. Harvesting electrical energy from the environment for electronic devices is important for researchers and engineers because modern electronic devices are characterized by low energy consumption [6,7,8]. Mechanical vibration energy harvesting is a method that could be very important in applications where there is no way to use wire connections to supply electricity or batteries to be replaced. To power small electronic devices and sensor energy harvesting from the surrounding environment using ambient energy sources, the design and deployment of the system are critical. In addition, batteries are inefficient and sometimes impossible to replace or recharge. Consequently, a lot of research has been conducted on energy harvesting technology as an independent source of power for portable devices or wireless sensor network systems. Energy harvesting technologies are already widely utilized, including wind, water, geothermal, and solar power plants [5,6,7,8,9]. The power generated from a piezoelectric transducer is significantly influenced by various factors, such as the applied load, the frequency of vibration, the geometric characteristics, and the boundary conditions. Mechanical vibrations occur in many practical locations, offering the advantage of being unrestricted by size or needing light [1,3,5,10,11]. The piezoelectric energy harvester is generally designed to operate in resonant mode to achieve the highest possible electrical power conversion. When functioning in this mode, the mechanical strain can become significant due to the substantial deformation at resonance. This paper focuses on mathematical and numerical modeling of an MFC piezoelectric energy harvester (PEH) system operating at resonance frequencies. The mechanical vibration energy harvester consists of a single MFC-P2 piezoelectric patch attached to an aluminum cantilever beam substrate with a rectangular cross-section. The design is anticipated to generate significant power over a resonance frequency range, making it suitable for low-frequency random vibration sources, such as those generated by human motion. Several ultra-low-power devices, such as low-power temperature sensors (TI’s LMT70 in sleep mode), low-power humidity sensors (which operate intermittently using sleep mode), nano-power real-time clocks (RTC) (Maxim DS3231 in standby), and piezoelectric MEMS vibration sensors (which can be self-powered by harvested vibration energy), use power from 0.1μW to 4.5μW [1,7,12,13]. The structure of this paper is organized as follows. The Introduction covers piezoelectric energy harvesting and the fundamental mechanisms of mechanical energy harvesting. Section 2 focuses on the mathematical modeling of electromechanical coupling and finite element modeling using COMSOL Multiphysics, specifically for direct MFC piezoelectric materials. Section 3 presents the results and discussion. In this section, the findings from both mathematical and numerical modeling are presented. The final section presents the conclusions and recommendations and summarizes the key points and implications of the study.

## 2. Materials and Methods

### 2.1. Piezoelectric Material, Selection, and Orientation

**Figure 1:**
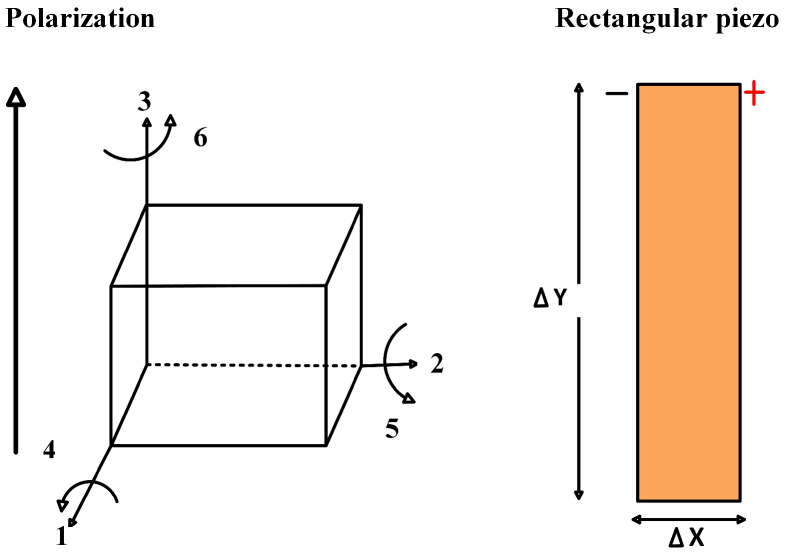
Directions and orientation axes of the piezoelectric material.

Piezoelectric properties differ for electrical/mechanical excitation along different directions. Once a piezoelectric material is chosen for a particular application, it is important to set the mechanical and electrical axes of operation. The orientation and poling axis strongly influence the piezoelectric effects. These axes are set during “poling”, which induces piezoelectric properties in the piezoelectric material. This polarization generates an electric field that can transform the mechanical energy used in the material’s deformation into electrical energy. The polarization generates an electric field, which causes the flow of the free charges in the conductor. In Figure 1, the piezoelectric material’s directions and orientation axes are shown. The axes X, Y, and Z represent the orthogonal coordinate system; axes 4, 5, and 6 are the axes of rotation. There are two commonly known piezoelectric modes of operation to harvest energy, i.e., the 3-1 loading mode $P_{2}$ and the 3-3 loading mode $P_{1}$ [10,11,14,15]. In the 3-1 loading mode, the direction of the electric field is perpendicular to the direction of the force applied. In the 3-3 mode, both the external stress and the generated voltage are aligned in the same direction [9,11,14,15].

(1) $$\mathrm{Piezoelectric}\;\mathrm{coefficient}\;\left(d\right)=\frac{\mathrm{Strain}\;\left(S\right)}{\mathrm{Electric}\;\mathrm{field}\;(E_{0})}.$$

(2) $$d_{33}=\frac{\Delta x/x}{\Delta v/x}=\frac{\Delta x\times x}{v\times x}=\frac{\Delta x}{v}.$$

(3) $$d_{31}=\frac{\Delta y/y}{v/v}=\frac{\Delta v\times x}{v\times y}.$$

Harvesting depends on various considerations, such as integration and compatibility, the material properties, the application, a dynamic analysis, the shape and size, the power requirements, and the frequency response. An MFC consists of a rectangular, uniaxially aligned piezo-fiber sandwiched between layers of adhesive and electrode polyimide films. The purpose of the rectangular fibers is to improve the contact between the piezoceramic and the adjacent electrode to ensure the efficient transfer of the electric field to the fibers [15,16]. An MFC can be designed with two distinct electrode patterns to operate in the $d_{33}$ mode or $d_{31}$ mode. The MFC is available in the contractor $d_{33}$ mode when polarization occurs along the length of the piezo-fiber (strain and electric field in the same direction) and elongates in $d_{31}$ when polarization occurs along the thickness of the piezo-fiber (strain induced in the z direction when an electric field is applied in the x direction). It consists of rectangular piezo-ceramic rods sandwiched between layers of adhesive, electrodes, and a polyimide film. Compared to $d_{31}$ mode, an MFC operating in $d_{33}$ mode has a lower electrical current but a higher energy conversion rate. In a $d_{31}$-type MFC, the applied voltages are mutually perpendicular to the PZT fiber direction, and, as a result, the transverse strain is obtained due to the coupling of the $d_{31}$ constant. For the $d_{33}$ mode of the MFC, the fiber orientation is along the longitudinal direction in alignment and longitudinal strain. The $d_{33}$ operating mode is expected to achieve higher performance, and, if a voltage is applied, it works as an actuator and will bend or distort materials, counteract vibrations, or generate vibrations. If no voltage is applied, it can work as a very sensitive strain gauge, sensing deformations, noise, and vibrations.

### 2.2. Modeling of Direct Piezoelectric Transducer for Energy Harvesting

**Figure 2:**
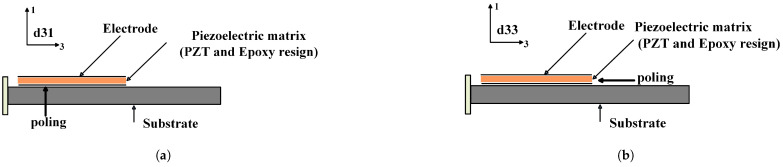
Orientation of piezoelectric materials with electrode polarization. (**a**) d31 Orientation. (**b**) d33 Orientation.

There are three primary mechanisms involved in converting mechanical energy into electrical energy: electromagnetic (EM), electrostatic (ES), and piezoelectric (PZ) [17]. (1) Electromagnetic (EM): This method is based on Faraday’s law of induction. Electromagnetic coils can produce higher power levels, ranging from 20 µW to 200 mW. However, they are generally larger, and the output voltages are often low, necessitating additional conversion stages. (2) Electrostatic (ES): Electrostatic devices utilize a type of variable capacitor to harvest energy, producing power levels between 10 and 20 µW. This power output is significantly lower than that of electromagnetic devices. Additionally, electrostatic devices require an external electrical source, such as a battery, to operate. (3) Piezoelectric (PZ): Piezoelectric transducers are advantageous for miniaturization, generating output voltages of up to 20 V. However, they have very high output impedances, resulting in output currents in the microampere range, which allows them to provide power levels of only a few tens of milliwatts. Piezoelectric energy harvesting technology utilizes the characteristic of electromechanical coupling of piezoelectric materials, directly converting mechanical energy to electrical energy, compared to electromagnetic and electrostatic generation. Direct piezoelectricity refers to the generation of an electrical field due to mechanical stress (acting as a sensor or generator), while reverse piezoelectricity describes the mechanical deformation caused by an external electrical field (functioning as an actuator or motor). In energy harvesting applications, the direct piezoelectric effect is utilized. This effect generates a voltage difference by displacing electrical charges through mechanical deformation. Figure 2 illustrates the electrode material orientation of piezoelectric materials for the energy harvesting method $d_{31}$ and $d_{33}$ respectively [15,18,19,20].

### 2.3. Mathematical Modeling of MFC Piezo-Transducer Integrated with Cantilever Beam

**Figure 3:**
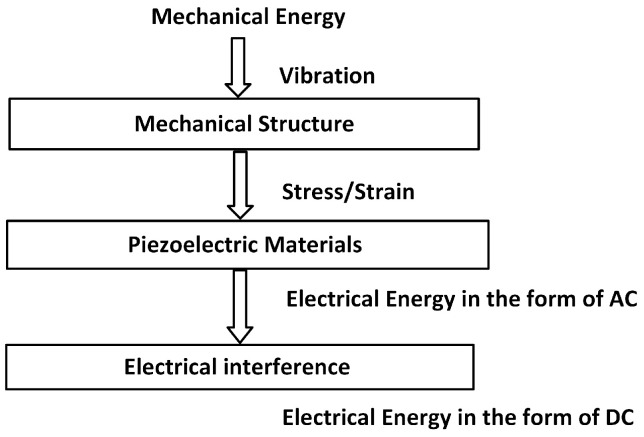
Piezoelectric energy harvesting flow charts.

In modeling an Energy harvester, it consists of piezoelectric materials, a mechanical structure and electrical energy interference. Mechanical structure uses due to input vibration; stress is developed over the surface of the piezoelectric materials. The output of electrical energy in the form of voltage is developed by these materials. In Figure 3 Energy Transducers, Energy Management and Energy Storage for several subsystems energy harvester to provide energy to the electronic application under suitable and stable voltage specification. Piezoelectric materials are continually being developed to create more effective transducers that enhance the piezoelectric properties and improve the efficiency of converting mechanical energy into electrical energy in energy harvesting applications. One example of an innovative piezoelectric transducer is the macro fiber composite (MFC) transducer, which was pioneered by NASA in 1996 [18,19,20,21,22]. The IEEE standard assumes that piezoelectric materials are linear. When poled piezoelectric materials are subjected to mechanical strain, they become electrically polarized, resulting in a fixed electric charge on the material’s surface (direct piezoelectric transducer).

Assuming linear characteristics in piezoelectric materials simplifies mathematical modeling and simulation. However, this assumption has several limitations, particularly when high accuracy is required in real-world energy harvesting applications. The energy density generated is directly proportional to the externally applied force. The mathematical relationship is expressed as follows:

(4) $$P_{pe}=d\times \sigma$$

where $P_{pe}$ represents the piezoelectric polarization vector (piezoelectric material under stress is polarized and surface charge appears), *d* represents the piezoelectric strain coefficient, and σ denotes the stress applied to the piezoelectric material. In the case of the reverse piezoelectric effect, the strain generated as a result is given by

(5) $$S_{pe}=d\times E_{0}.$$

The direct and reverse piezoelectric effects can be expressed in alternative formulations as follows:

(6) $$P_{pe}=d\times \sigma =d\times s\times E.$$

(7) $$\sigma_{pe}=E\times S_{pe}=E\times d\times E_{0}=e\times E_{0}$$

The relationship between material polarization and deformation can be defined in two forms: the strain–charge form and the stress–charge form. Now, we add Equation (5) to Equation (4) and Equation (7) to Equation (6).

Strain–charge equation:

(8) $$\left\{\begin{array}{c} S=s^{E}\sigma +d^{t}E\\ D=d\sigma +\varepsilon^{T}E. \end{array}\right.$$

where *S* is the mechanical strain (6 × 1) vector, in m/m; σ is the mechanical stress (6 × 1) vector, in Pa; *D* is the electric displacement (3 × 1) vector, in C/m^2^; *E* is the electric field (3 × 1) vector, in V/m; $s^{E}$ is the compliance matrix at a constant electric field (6 × 6), in 1/Pa; *d* is the piezoelectric charge coefficient matrix (3 × 6), in C/N or m/V; $\epsilon^{T}$ is the permittivity matrix at constant stress (3 × 3), in F/m; and $d^{t}$ is the transpose of the *d* matrix.

Stress–charge form:
  (9) $$\sigma_{ij}=C_{ijkl}s-e_{kij}E_{o_{k}},$$
  (10) $$D_{i}=e_{ijk}s_{jk}+s_{ij}^{\epsilon }E_{o_{k}}.$$

Stress–voltage form:

(11) $$\sigma =C^{D}s-h^{t}D,$$

(12) $$E=-hs+s^{\epsilon -1}D.$$

The equations above can be written in matrix form:

(13) $$\left[\begin{array}{c} s_{1}\\ s_{2}\\ s_{3}\\ s_{4}\\ s_{5}\\ s_{6}\\ D_{1}\\ D_{2}\\ D_{3} \end{array}\right]=\left[\begin{array}{ccccccccc} S_{11} & S_{12} & S_{13} & S_{14} & S_{15} & S_{16} & d_{11} & d_{21} & d_{31}\\ S_{21} & S_{22} & S_{23} & S_{24} & S_{25} & S_{26} & d_{12} & d_{22} & d_{32}\\ S_{31} & S_{32} & S_{33} & S_{4} & S_{35} & S_{36} & d_{13} & d_{23} & d_{33}\\ S_{41} & S_{42} & S_{43} & S_{44} & S_{45} & S_{46} & d_{14} & d_{24} & d_{34}\\ S_{51} & S_{52} & S_{53} & S_{54} & S_{55} & S_{56} & d_{15} & d_{25} & d_{35}\\ S_{61} & S_{62} & S_{63} & S_{64} & S_{65} & S_{66} & d_{16} & d_{26} & d_{36}\\ d_{11} & d_{12} & d_{13} & d_{14} & d_{15} & d_{16} & \epsilon_{11}^{\sigma } & \epsilon_{12}^{\sigma } & \epsilon_{13}^{\sigma }\\ d_{21} & d_{22} & d_{23} & d_{24} & d_{25} & d_{26} & \epsilon_{21}^{\sigma } & \epsilon_{22}^{\sigma } & \epsilon_{23}^{\sigma }\\ d_{31} & d_{32} & d_{33} & d_{34} & d_{35} & d_{36} & \epsilon_{31}^{\sigma } & \epsilon_{2}^{\sigma } & \epsilon_{33}^{\sigma } \end{array}\right]\left[\begin{array}{c} \sigma_{1}\\ \sigma_{2}\\ \sigma_{3}\\ \sigma_{4}\\ \sigma_{5}\\ \sigma_{6}\\ E_{0_{1}}\\ E_{0_{2}}\\ E_{0_{3}} \end{array}\right].$$

In the case of non-piezoelectric materials, the constitutive equation is reduced to only pure mechanical stress–strain:

(14) σ=S×E.

For one-dimensional piezoelectric materials poled along their thickness, the $d_{31}$ polarization aligns with direction 3, while stress is applied along direction 1. The piezoelectric constitutive equations can be defined for strain–charge and stress–charge as follows:

(15) $$\left\{\begin{array}{c} S_{1}=s_{11}^{E}\sigma_{1}+d_{31}E_{3}\\ D_{3}=d_{31}\sigma_{1}+\epsilon_{33}^{T}E_{3} \end{array}\right.$$

(16) $$\left\{\begin{array}{c} \sigma_{xx}^{p}=E_{p}s_{xx}^{p}-e_{31}E_{z}\\ D_{z}=e_{31}s_{xx}^{p}+\epsilon_{33}E_{z} \end{array}\right.$$

where $s_{11}^{E}\sigma_{1}$ is the strain due to mechanical stress (Hooke’s law) in the axial direction; $d_{31}E_{3}$ is the strain induced by the electric field (converse piezoelectric effect) in axial strain; $d_{31}\sigma_{1}$ is the charge generated due to mechanical stress (direct piezoelectric effect); $\epsilon_{33}^{T}E_{3}$ is the electric displacement due to the electric field; $D_{3}$ is the electric displacement in the polarization direction; $e_{31}$ is the piezoelectric coupling coefficient; and $E_{3}$ is the electric field in the thickness (polarization direction).

The full constitutive equations in matrix form for transverse vibration when polarization occurs along the z-axis and strain occurs in the x-axis direction are

(17) $$\left\{\begin{array}{c} \left[\begin{array}{c} S_{1}\\ S_{2}\\ S_{3}\\ S_{4}\\ S_{5}\\ S_{6} \end{array}\right]=\left[\begin{array}{cccccc} s_{11} & s_{12} & s_{13} & 0 & 0 & 0\\ s_{12} & s_{22} & s_{23} & 0 & 0 & 0\\ s_{13} & s_{23} & s_{33} & 0 & 0 & 0\\ 0 & 0 & 0 & s_{44} & 0 & 0\\ 0 & 0 & 0 & 0 & s_{55} & 0\\ 0 & 0 & 0 & 0 & 0 & s_{66} \end{array}\right]\left[\begin{array}{c} \sigma_{1}\\ \sigma_{2}\\ \sigma_{3}\\ \sigma_{4}\\ \sigma_{5}\\ \sigma_{6} \end{array}\right]+\left[\begin{array}{ccc} d_{11} & d_{21} & d_{31}\\ d_{12} & d_{22} & d_{32}\\ d_{13} & d_{23} & d_{33}\\ d_{14} & d_{24} & d_{34}\\ d_{15} & d_{25} & d_{35}\\ d_{16} & d_{26} & d_{36} \end{array}\right]\left[\begin{array}{c} E_{1}\\ E_{2}\\ E_{3} \end{array}\right]\\ \left[\begin{array}{c} D_{1}\\ D_{2}\\ D_{3} \end{array}\right]=\left[\begin{array}{cccccc} d_{11} & d_{12} & d_{13} & d_{14} & d_{15} & d_{16}\\ d_{21} & d_{22} & d_{23} & d_{24} & d_{25} & d_{26}\\ d_{31} & d_{32} & d_{33} & d_{34} & d_{35} & d_{36} \end{array}\right]\left[\begin{array}{c} \sigma_{1}\\ \sigma_{2}\\ \sigma_{3}\\ \sigma_{4}\\ \sigma_{5}\\ \sigma_{6} \end{array}\right]+\left[\begin{array}{ccc} \epsilon_{11} & 0 & 0\\ 0 & \epsilon_{22} & 0\\ 0 & 0 & \epsilon_{33} \end{array}\right]\left[\begin{array}{c} E_{1}\\ E_{2}\\ E_{3} \end{array}\right]. \end{array}\right.$$

**Figure 4:**
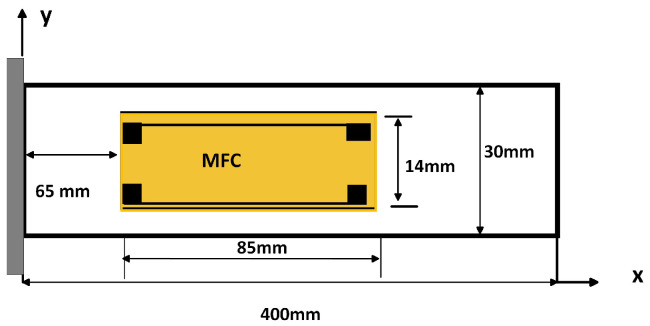
Geometry of cantilever beam bonded with unimorph MFC piezoelectric material.

The theoretical or analytical modeling of a cantilever beam using unimorph macro fiber composites (MFC) as a piezoelectric material is based on Euler–Bernoulli’s theory [15,17]. In this theory, rotating inertia and shear deformations are considered negligible, and it assumes that the beam’s length is significantly greater than its thickness and width. It is also assumed that there is perfect bonding between the MFC layer and the beam element as shown in Figure 4 below. Both the top and bottom surfaces of the MFC are covered with copper electrodes, designated as the ground and terminal, respectively. The system operates in the $d_{31}$ mode, indicating that the piezoelectric layer is polarized in the 3-direction. Consequently, the electrical charge generated will discharge from the electrodes in the 3-direction, while strain occurs along the 1-axis. Thin beam theory applies to beams whose length is much greater than their depth. In the case of the transverse forced vibration of a cantilever beam with a unimorph MFC piezoelectric material, the output power is influenced by several factors, including the beam’s geometry, the excitation frequency, the magnitude of the externally applied force, the material properties, and the boundary conditions. According to Euler’s theory, the governing equation for the transverse vibration of a cantilever beam with a unimorph MFC is [9,15,17,18,19,20].

(18) $$\rho_{eq}\frac{d^{2}w\left(x,t\right)}{dt^{2}}+EI_{eq}\frac{\partial^{4}w\left(x,t\right)}{\partial x^{4}}=-P\left(t\right)\delta \left(L-x_{F}\right)+F_{\mathrm{piezo}.}$$

where the deflection of the beam at position x and time *t* is represented as w(x,t); EI is the flexural rigidity of the beam (including the MFC layer); c is the damping coefficient; ρ is the mass per unit length of the beam (including the MFC layer); F(t) is the dynamic force applied at the free end (x=L); δ(x−L) is the Dirac delta function representing the point load at the free end; and $F_{\mathrm{piezo}}$ is the piezoelectric force generated by the MFC [21,22,23].

The strain throughout the beam thickness in a Euler–Bernoulli-modeled beam is proportional to the distance from the neutral axis. Using constitutive equations for piezoelectric materials, the strain ‘S’ in the beam is at a distance y from the neutral axis (where there is no elongation or compression):

(19) $$s_{x}=y\frac{\partial^{2}w\left(x,t\right)}{\partial x^{2}}=h_{p}\frac{\partial^{2}w\left(x,t\right)}{\partial x^{2}}.$$

The stress ‘σ’ in the MFC is proportional to the strain ‘S’:

(20) $$\sigma =E_{p}\times S=-E_{p}\times h_{p}\times \frac{\partial^{2}w\left(x,t\right)}{\partial x^{2}}$$

where $E_{p}$ denotes the Young’s modulus of the MFC.

From Equation (19), we can observe that the macro fiber composite (MFC) is bonded to the top surface of the beam. Consequently, the distance *y* from the neutral axis to the center of the MFC is referred to as $h_{p}$. The total stress in the MFC connected to the cantilever beam is composed of two components: the piezoelectric stress resulting from the piezoelectric effect and the mechanical stress due to bending. Since the MFC patch is perfectly bonded to the surface of the beam, the piezoelectric effect can be considered linear and is governed by the constitutive equations. This piezoelectric coupling introduces an additional term in the bending moment equation that is related to the generated voltage. The electrical circuit equation will incorporate the capacitance of the MFC and the load resistance. Given that the electrodes on the opposing faces of the MFC layer are significantly thin compared to the overall thickness of the harvester, their contribution to the thickness dimension can be considered negligible, along with the adhesive layer. To perform analytical and numerical simulations to derive the electromechanical coupling equations of piezoelectric materials, the following assumptions are made based on the figure above.

- The piezoelectric layer electrodes and the adhesive layer have a negligible thickness.
- There is perfect bonding between the piezoelectric layer and the substrate layers.
- A constant, uniform electric field exists throughout the thickness of the piezoelectric layer.
- Proportional damping is present across all harvester configurations.
- The layer topology is rectangular, maintaining a constant width and thickness [24,25,26,27,28,29,30,31,32,33,34,35].

(21) $$\sigma_{x}=c_{11}\frac{\partial^{2}w\left(x,t\right)}{\partial x^{2}}h_{p}-e_{31}E_{3}.$$

The electric field *E* within the MFC is related to the voltage V(t) applied across it and its thickness, based on the piezoelectric constitutive equations (Equation (16)). For this analysis, we assume that the electric field is uniform across the thickness of the MFC. This is illustrated in the figure above.

(22) $$E=\frac{V(t)}{h_{p}}.$$

The final equation for the total stress becomes

(23) $$\sigma_{x}=c_{11}\frac{\partial^{2}w\left(x,t\right)}{\partial x^{2}}h_{p}-e_{31}\frac{V(t)}{h_{p}}.$$

The neutral axis $\bar{y}$ of the composite beam and MFC section is defined as

(24) $$\bar{y}=\frac{E_{b}A_{b}\frac{h_{b}}{2}+E_{p}A_{p}\left(h_{b}+\frac{h_{p}}{2}\right)}{2(E_{b}A_{b}+E_{p}A_{p})}).$$

where $A_{b}=b_{b}h_{b}$ is the beam cross-section and $A_{p}=b_{p}h_{p}$ is the MFC cross-section.

Effective flexural rigidity:

(25) $${\left(EI\right)}_{\mathrm{comp}}=E_{b}\left[I_{b}+A_{b}{\left(\bar{y}-\frac{h_{b}}{2}\right)}^{2}\right]+E_{p}\left[I_{p}+A_{p}{\left(h_{b}+\frac{h_{p}}{2}-\bar{y}\right)}^{2}\right],$$

where $I_{b}=\frac{b_{b}h_{b}^{3}}{12},I_{p}=\frac{b_{p}h_{p}^{3}}{12}$

The force generated by the piezoelectric material is obtained using the piezoelectric material’s constitutive equation $\sigma_{x}=c_{11}\epsilon_{x}+e_{31}E_{3}$, $\epsilon_{x}=\frac{\partial^{2}w\left(x,t\right)}{\partial x^{2}}h_{p}$ and $0\leq x_{1}\leq x_{2}\leq L$.

(26) $$F_{\mathrm{piezo}}\left(x,t\right)=\frac{\partial }{\partial x}\left[bh_{p}\left(c_{11}h_{p}\frac{\partial^{2}w\left(x,t\right)}{\partial x^{2}}+e_{31}h_{p}v\left(t\right)\right)\right]=bh_{p}^{2}c_{11}\frac{\partial^{3}w\left(x,t\right)}{\partial x^{3}}+bh_{p}e_{31}\frac{\partial v(t)}{\partial x},$$

where

- *b*: width of the piezoelectric layer;
- $h_{p}$: thickness of the piezoelectric layer;
- $c_{11}$: elastic modulus of the piezoelectric layer;
- $e_{31}$: piezoelectric stress constant;
- v(t): voltage across the piezoelectric layer;
- $x_{1}$ and $x_{2}$: positions along the beam where the macro fiber composite (MFC) is attached.

The piezoelectric coupling equation is a partial differential equation that governs the mechanical dynamics of a beam when it is integrated with an MFC piezoelectric transducer. It is

(27) $$m\frac{d^{2}w}{dt^{2}}+EI\frac{\partial^{4}w}{\partial x^{4}}=-P\left(t\right)\delta \left(L-x_{F}\right)+bh_{p}^{2}c_{11}\frac{\partial^{3}w\left(x,t\right)}{\partial x^{3}}+bh_{p}e_{31}\frac{\partial v(t)}{\partial x},$$

where $c_{11}h_{p}\frac{\partial^{2}w\left(x,t\right)}{\partial x^{2}}$ is the mechanical stress contribution and $e_{31}h_{p}v\left(t\right)$ is the the electric field-induced stress due to the MFC voltage.

After rearrangement, the governing equation of the beam and piezoelectric coupling is

(28) $$\begin{array}{c} \rho_{\mathrm{eq}}\frac{\partial^{2}w\left(x,t\right)}{\partial t^{2}}+\frac{\partial^{2}}{\partial x^{2}}\left(EI_{\mathrm{eq}}\frac{\partial^{2}w\left(x,t\right)}{\partial x^{2}}\right)=\frac{\partial }{\partial x}\left[bh_{p}\left(c_{11}h_{p}\frac{\partial^{2}w\left(x,t\right)}{\partial x^{2}}+e_{31}h_{p}v\left(t\right)\right)\right]\\ -P\left(t\right)\delta \left(L-x_{F}\right), \end{array}$$

where $\rho_{eq}$ is the equivalent mass density per unit length of the beam (including the piezoelectric layer), $EI_{eq}$ is the equivalent flexural rigidity of the beam and piezoelectric layer, $F_{piezo}\left(x,t\right)$ is the force due to piezoelectric effects (above), $P(t)\delta (L-x_{F})$ is the Dirac delta function concentrated transverse vibration force applied to the free end, and w(x,t) is the transverse displacement of the beam. The effective mass per unit length of the composite beam is

(29) $$\rho_{\mathrm{eq}}=\rho_{\mathrm{beam}}A_{\mathrm{beam}}+\rho_{\mathrm{piezo}}A_{\mathrm{piezo}},$$

where $\rho_{\mathrm{beam}}$ is the density of the beam material, $A_{\mathrm{beam}}=bh$ is the cross-sectional area of the host beam, $\rho_{\mathrm{piezo}}$ is the density of the piezoelectric material, and $A_{\mathrm{piezo}}$ is the cross-sectional area of the piezoelectric layer.

The stiffness of the composite beam combines contributions from the host beam and the piezoelectric layer:

(30) $${\mathrm{EI}}_{\mathrm{eq}}=E_{\mathrm{beam}}I_{\mathrm{beam}}+E_{\mathrm{piezo}}I_{\mathrm{piezo}},$$

where $E_{\mathrm{beam}}$ is the Young’s modulus of the beam material, $I_{\mathrm{beam}}=\frac{bh^{3}}{12}$ is the moment of inertia of the host beam, $E_{\mathrm{piezo}}$ is the Young’s modulus of the piezoelectric layer, and $I_{\mathrm{piezo}}$ is the moment of inertia of the piezoelectric layer (which depends on the layer geometry and position).

When modeling the electrical equations, it should be noted that only the electrical displacement in the z (or 3) direction is non-zero due to the poling direction and the strain in x (or 1). For electric circuit (electrical) dynamics, the charge generated in the MFC layer is due to the piezoelectric effect, which causes the MFC to produce a voltage (or electric potential) when subjected to mechanical deformation. This deformation comes from bending the beam under an externally applied load. The total charge Q(t) on the electrodes of the MFC is

(31) $$Q\left(t\right)=\int_{\mathrm{electrode}\;\mathrm{area}}D_{3}\;dA,$$

(32) $$Q\left(t\right)=\int_{x_{1}}^{x_{2}}\int_{0}^{b}D_{3}\;dz\;dx.$$

Since bending-induced axial strain in the x-direction (1-direction) is the only source of electrical output, the tensor-based piezoelectric constitutive Equation (13) describing the electric displacement vector can be simplified to a scalar equation, where $D_{3}=\epsilon_{33}E_{3}+e_{31}\epsilon_{x}$; here, $E_{3}=-\frac{v(t)}{h_{p}}$ is the electric field across the MFC, and $\epsilon_{x}=h_{p}\frac{\partial^{2}w\left(x,t\right)}{\partial x^{2}}$ represents the strain in the MFC.

Using Equations (14), (20) and (22), $D_{3}$ is as follows:

(33) $$D_{3}(x,\;t)=-E_{p}d_{31}t_{\mathrm{pc}}\frac{\partial^{2}w\left(x,t\right)}{\partial x^{2}}-\frac{\varepsilon_{33}^{S}}{h_{p}}V(t).$$

We substitute for $D_{3}$

(34) $$Q\left(t\right)=\int_{x_{1}}^{x_{2}}b\left[\epsilon_{33}h_{p}v\left(t\right)+e_{31}h_{p}\frac{\partial^{2}w\left(x,t\right)}{\partial x^{2}}\right]dx.$$

From the relations of the current (I), charge (Q), voltage (v), resistance load ($R_{1}$), and capacitance ($C_{p}$),

(35) $$I\left(t\right)=\frac{dQ(t)}{dt}=\frac{v(t)}{R_{1}}=C_{p}\frac{dV_{c}\left(t\right)}{dt}=-\int_{x_{1}}^{x_{2}}E_{p}d_{31}t_{pc}b_{p}\frac{\partial^{3}w\left(x,t\right)}{\partial x^{2}\partial t}\;dx-C_{p}\frac{\partial V(t)}{\partial t}.$$

After differentiation and rearrangement, the electrical circuit equation for the charge on the MFC is

(36) $$\begin{array}{cc} c_{p}\frac{d}{dt}v\left(t\right)+R_{l}v\left(t\right)+e_{31}bh_{p}\int_{x_{1}}^{x_{2}}\frac{\partial^{3}w\left(x,t\right)}{\partial x^{2}\partial t}\;dx & =0\\ or\\ \;\frac{V_{R}\left(t\right)}{R_{l}}+\frac{{\bar{\varepsilon }}_{33}b_{p}L_{p}}{h_{p}c_{p}}\frac{dV_{R}\left(t\right)}{dt} & =-bh_{p}e_{31}\int_{x_{1}}^{x_{2}}\left(\frac{dw_{n}\left(x\right)}{dx}\right)\eta_{n}\left(t\right) \end{array}$$

where $c_{p}$ = $\frac{\varepsilon_{33}^{S}b_{p}\left(x_{2}-x_{1}\right)}{h_{p}}$ is the capacitance of the piezoelectric layer, $\frac{dv(t)}{dt}$ is the time derivative of the voltage generated across the MFC layer, $R_{l}$ is the load resistance in the electrical circuit, and v(t) is the voltage across the MFC layer. The term $e_{31}\cdot bh_{p}$ is a scaling factor related to the piezoelectric coefficient and the MFC geometry, while $\int_{x_{1}}^{x_{2}}\frac{\partial^{2}w\left(x,t\right)}{\partial x^{2}}\;dx$ represents the integrated curvature of the beam over the region where the MFC is applied (from $x_{1}$ to $x_{2}$). According to Equation (36), the voltage generated by the piezoelectric transducer is determined by the beam’s mode response, mode shape, internal capacitance, and load resistance and the material’s mechanical and electrical properties. From Equation (36), let us say that $\alpha =\frac{\epsilon_{33}\left(x_{2}-x_{1}\right)bh_{p}}{R_{l}}$ represents the damping effect of the resistive load and $\beta =-\epsilon_{33}e_{31}$ denotes the beam deformation and curvature changes to the electrical output.

The final equation is

(37) $$\frac{d}{dt}v(t)+\alpha v(t)=\beta \int_{x_{1}}^{x_{2}}\frac{\partial^{3}w(x,\;t)}{\partial x^{2}\;\partial t}\;\mathrm{dx}.$$

To model direct piezoelectric energy harvesting, the piezoelectric element is coupled with an external circuit. It exhibits capacitive behavior due to the piezoelectric material and resistive behavior from the electrical load.

(38) $$V_{p}\left(t\right)=V_{c}\left(t\right)+R_{z}C_{p}\frac{d}{dt}V_{c}\left(t\right)=\frac{d_{31}\cdot \omega_{n}\cdot X}{{\left(\omega_{n}C_{p}\right)}^{2}+{\left(1/R\right)}^{2}},$$

where $V_{p}\left(t\right)$ is the total voltage generated by the piezoelectric element, $V_{c}\left(t\right)$ is the voltage across the capacitive element of the piezoelectric material, and $R_{z}C_{p}\frac{dV_{c}\left(t\right)}{dt}$ is the additional voltage component due to the dynamic behavior of the charge and its flow through the resistive element $R_{z}$. The power output of a piezoelectric mechanical energy harvester is a function of the load. The following formula gives the relationship for the electrical power (P) dissipated in a resistive load:

(39) $$P_{\mathrm{out}}=\frac{V^{2}}{R_{l}}.$$

#### Natural Frequency and Mode Shape

Modal analysis is performed to identify mode shapes and resonant frequencies. The natural frequency is the frequency at which a system oscillates when displaced from its equilibrium position and allowed to vibrate freely. For a cantilever beam, the natural frequencies depend on its material properties, geometry, and boundary conditions. The mode shape describes the deformation pattern of the beam at a specific natural frequency. This shape represents the beam’s vibrational configuration when oscillating at its natural frequency, with each natural frequency corresponding to a unique mode shape. For free vibration, the external excitation is assumed to be zero (P(t)$\delta \left({L\text{-}X}_{F}\right)+P_{\mathrm{piezo}}=\;0$); Equation (21) is simplified to

(40) $$m\frac{d^{2}w}{dt^{2}}+EI\frac{\partial^{4}w}{\partial x^{4}}=0,$$

(41) $$C=\sqrt{\frac{EI}{m}}=\sqrt{\frac{EI}{\rho A}}.$$

For the transverse vibration of a cantilever beam integrated with an MFC, Equation (40) is solved by the method of separation of variables:

(42) w(x,t)=ϕ(x)×η(t)

where ϕ(x) represents the spatial component and η(t) represents the temporal component. Using Equations (40)–(42) and rearranging them yields

(43) $$\frac{c^{2}}{W(x)}\frac{d^{4}W\left(x\right)}{dx^{4}}=-\frac{1}{\eta (t)}\frac{d^{2}\eta \left(t\right)}{dt^{2}}=b=\omega^{2},$$

(44) $$\frac{d^{4}W\left(x\right)}{dx^{4}}-\alpha^{4}W\left(x\right)=0.$$

The equation for the temporal component is

(45) $$\frac{d^{2}\eta \left(t\right)}{dt^{2}}+\omega^{2}\eta \left(t\right)=0.$$

The curvature and transverse displacement of a beam can be determined using the fundamental Euler–Bernoulli beam equation, considering the specified boundary conditions. The power harvester consists of an MFC $d_{31}$ piezoelectric unimorph clamped to an aluminum cantilever beam at 65 mm from a fixed support.

The boundary conditions for a cantilever beam with a fixed support x=0 and a free end $x=L_{\mathrm{eff}}$ are as follows.

x=0 (fixed end): $w\left(0,t\right)=0\;\left(\mathrm{no}\mathrm{displacement}\right),\;\frac{\partial w(0,\;t)}{\partial x}=0\;\left(\mathrm{no}\mathrm{slope}\right)$
$x=L_{\mathrm{eff}}$ (free end): $\frac{\partial^{3}\;w\left(L_{\mathrm{eff}},t\right)}{\partial x^{3}}=0\;\left(\mathrm{no}\mathrm{shear}\mathrm{force}\right),\;\frac{\partial^{2}w\left(L_{\mathrm{eff}},t\right)}{\partial x^{2}}=0\;\left(\mathrm{no}\mathrm{bending}\mathrm{moment}\right).$

These boundary conditions give rise to a transcendental equation, which yields the characteristic values of αL = 1.875, 4.694, 7.855, …

The solution equation of the mode shape function (44) is in exponential form:

(46) $$W\left(x\right)=De^{bx}$$

where *a a a D* and *b* are constants.

After the substitution of (46) into Equation (44),

(47) $$b^{4}-\alpha^{4}=0.$$

Equation (46) becomes

(48) $$b_{1,2}=\pm \alpha ,\;b_{3,4}=\pm i\alpha ,$$

where α is constant and *i* is the imaginary unit. The values of *b* are as follows: $b_{1,2}=\pm \beta$ represents the values $b_{1}$ and $b_{2}$, which are ±α, and $b_{3,4}=\pm i\alpha$ represents the values $b_{3}$ and $b_{4}$, which are ±iα, where *i* is the imaginary unit. The final solution of Equation (29) can be expressed as

(49) $$W\left(x\right)=D_{1}e^{\alpha x}+D_{2}e^{-\alpha x}+D_{3}e^{i\alpha x}+D_{4}e^{-i\alpha x},$$

where α is a parameter related to the natural frequencies obtained from the boundary conditions and depends on the mode number n, and the constants $D_{1},D_{2},D_{3},D_{4}$ are determined by the boundary conditions.

Alternatively,

(50) $$\begin{array}{cc} W(x) & =D_{1}\left(\cos\alpha x+\cosh\alpha x\right)+D_{2}\left(\cos\alpha x-\cosh\alpha x\right)\\ \; & \;+D_{3}\left(\sin\alpha x+\sinh\alpha x\right)+D_{4}\left(\sin\alpha x-\sinh\alpha x\right). \end{array}$$

The relationship between α and the angular natural frequency ω is

(51) $$\omega_{n}=\frac{\alpha_{n}^{2}}{L_{\mathrm{eff}}^{2}}\sqrt{\frac{\rho A}{EI}}.$$

For a cantilever beam with an effective length $\left(L_{e}ff\right)$, effective properties for beams and MFCs in terms of the effective modulus, density, area, and moment of inertia can be determined with Equation (52) for any mode n,

(52) $$\omega_{n}=\frac{\alpha_{n}^{2}}{L_{\mathrm{eff}}^{2}}\sqrt{\frac{\rho_{eff}A_{eff}}{E_{eff}I_{eff}}},$$

where ($E_{\mathrm{eff}}$) is the weighted modulus based on the stiffness contributions of the materials; ($\rho_{\mathrm{eff}}$) is the weighted density based on the mass contributions of the materials; ($A_{\mathrm{eff}}$) is the sum of the cross-sectional areas; and ($I_{\mathrm{eff}}$) is the moment of inertia for the composite cross-section, considering the parallel axis theorem. The solution of Equation (40) is

(53) η(t)=Acos(ωt)+Bsin(ωt),

where A and B are constants that can be found from the initial conditions.

From Figure 4, for the natural frequencies and mode shapes of beams at a fixed support x=0 and free at x=L, the boundary conditions are as follows:

W(0)=0, $\frac{dW}{dx}\left(0\right)=0$, $EI\frac{d^{2}W}{dx^{2}}\left(l\right)=0$ or $\frac{d^{2}W}{dx^{2}}\left(l\right)=0$ and $EI\frac{d^{3}W}{dx^{3}}\left(l\right)=0$ or $\frac{d^{3}W}{dx^{3}}\left(l\right)=0$.

When these boundary conditions are used in (50),

(54) $$D_{1}+D_{3}=0.$$

Moreover,

(55) $$D_{2}\left(\cos\alpha L+\cosh\alpha L\right)+D_{4}\left(\sin\alpha L+\sinh\alpha L\right)=0,$$

(56) $$D_{2}\left(-\sin\alpha L+\sinh\alpha L\right)+D_{4}\left(\cos\alpha L+\cosh\alpha L\right)=0.$$

From Equation (56),

(57) $$D_{4}=-\frac{\cos\alpha L+\cosh\alpha L}{\sin\alpha L+\sinh\alpha L}D_{2}.$$

The combination of Equations (56)–(58) leads to natural frequencies

(58) cosαLcoshαL+1=0.

Convert $\omega_{n}$ to the natural frequency $f_{n}$ in Hz for any mode n (Equation (53)):

(59) $$f_{n}=\frac{\omega_{n}}{2\pi }=\frac{\alpha_{n}^{2}}{2\pi }\sqrt{\frac{\rho A}{EI}}={\left(\alpha_{n}l^{2}\frac{EI}{\rho AL^{4}}\right)}^{1/2}=\frac{\pi^{2}n^{2}}{L_{\mathrm{eff}}^{2}}\sqrt{\frac{\rho_{\mathrm{eff}}A_{\mathrm{eff}}}{E_{\mathrm{eff}}I_{\mathrm{eff}}}},$$

where $\alpha_{n}l$ = $\frac{(2n-1)\pi }{2}$, *n* is an integer, *E* is the modulus of elasticity, *I* is the second moment of the area, ρ is the density, *A* is the cross-sectional area, and *L* is the length of the beam.

The nth mode shape can be expressed as

(60) $$W_{n}\left(x\right)=\left(\cos\alpha_{n}x-\cosh\alpha_{n}x\right)-\frac{\cos\alpha_{n}L+\cosh\alpha_{n}L}{\sin\alpha_{n}L+\sinh\alpha_{n}L}\left(\sin\alpha_{n}x-\sinh\alpha_{n}x\right).$$

The piezoelectric layer couples the electrical and mechanical responses; the source of mechanical strain is assumed to be the axial strain due to bending for the given electrode configuration.

### 2.4. Numerical Modeling Using COMSOL Multiphysics

**Figure 5:**
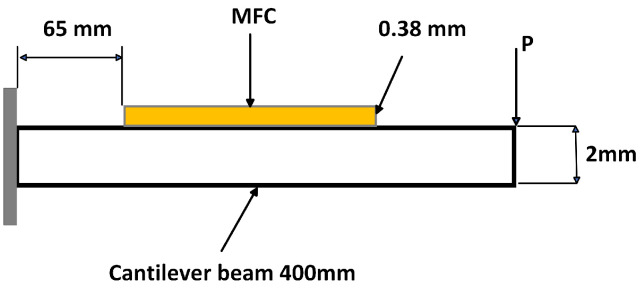
MFC integrated with Al cantilever beam fixed at one end and free at the other.

The aluminum cantilever beam is bonded with unimorph MFC piezoelectric materials and modeled using the 3D COMSOL version 6.29. One of the cantilever ends has a fixed boundary condition, while the other ends are assigned free boundary conditions. As shown in Figure 5, the M8514-P2 PE patch is bonded to the upper side of the cantilever beam at a length of 65 mm from the fixed support, which has a rectangular cross-section. The P2-type macro fiber composites ($\mathrm{MFC}-P_{2}$) are specifically designed for energy harvesting applications. They offer high electric source capacitance and enhanced electric charge generation. They are constructed from piezoelectric fibers and are environmentally sealed, resulting in greater flexibility and durability compared to standard monolithic piezo-ceramic harvesters. The constitutive equations of a piezoelectric material enable electromechanical coupling in the multiphysics structure. The strain–charge form, available in COMSOL Multiphysics, is adopted since it drastically reduces the number of material constants when some assumptions are made. A point load at the free end of the cantilever beam, which has the average force of 0.2–2 N, is applied to the top of the aluminum beam, and the power across the top and bottom of the patch is computed when the load resistance is 12,000 ohm [6,35,36,37,38,39,40,41,42,43,44].

## 3. Results and Discussion

### 3.1. Component Parameters, Material Properties, and Dimensions for Analytical and Numerical Energy Harvester Model

The aluminum substrate beam, PZT-5A, MFC piezoelectric transducer, epoxy resin, and copper electrodes are used for geometry construction; their nominal dimensions, as well as some physical and electromechanical properties of the energy harvester used in this study, are listed in Table 1 below.

### 3.2. Analytical Design: Natural Frequency and Mode Shape

The MFC placed from x = 65 mm to 150 mm will experience varying strain. From Equation (20) and Figure 5, the maximum strain is at the fixed end (x = 0), and it decreases linearly to zero at the free end (for a static point load). To compute the average strain over the MFC’s length, we integrate the strain distribution over this interval. From Euler–Bernoulli beam theory, the strain at any point in the beam depends on the distance from the neutral axis and the curvature of the beam. Due to transverse vibrations, the beam is dynamically loaded, which means that the deflection w depends on position x. The second moment of area (I) for the composite structure (beam + MFC) is derived from the theorem of the parallel axis using Equation (24). The neutral axis (NA) position can be calculated by finding the centroid of the transformed cross-section.

$$h_{\mathrm{Al}\text{-}\mathrm{MFC}}=\frac{A_{\mathrm{Al}}h_{\mathrm{Al}}+A_{{\mathrm{MFC}}_{\mathrm{transformed}}}h_{\mathrm{MFC}}}{A_{\mathrm{Al}}+A_{{\mathrm{MFC}}_{\mathrm{transformed}}}}=1.097\;\mathrm{mm}.$$

Thus, the NA shifts up slightly by 0.097 mm due to the MFC.

The second moment of area $I_{\mathrm{total}}$ is the sum of the individual Is plus the parallel axis term.

For the aluminum layer, $I_{\mathrm{Al}}=\left(30\times 2^{3}\right)/12+30\times 2\times {\left(1-1.097\right)}^{2}=\left(30\times 8\right)/12+30\times 2\times {\left(0.097\right)}^{2}=20.564\;{\mathrm{mm}}^{4}$

For the transformed MFC layer, $I_{\mathrm{MFC}}=\frac{8.4\times 0.38^{3}}{12}+8.4\times 0.38\times {\left(2.19\;-\;1.097\right)}^{2}=6.414$ mm^4^.

Total $I_{\mathrm{total}}=20.564+6.414=26.978\;{\mathrm{mm}}^{4}=26.978\times 10^{-12}\;m^{4}$.

$$E_{\mathrm{Al}}I_{\mathrm{Al}}=\left(70\times 10^{9}\right)\times \left(20.564\times 10^{-12}\right)=1.412\;{\mathrm{Nm}}^{2}$$

and

$$E_{\mathrm{MFC}}I_{\mathrm{MFC}}=37.8\times 10^{9}\times 6.414\times 10^{-12}=0.261\;{\mathrm{Nm}}^{2}$$

Thus, in the region with the MFC, ${\mathrm{EI}}_{\mathrm{Composite}}=E_{\mathrm{Al}}\times I_{\mathrm{Al}}+E_{\mathrm{MFC}}\times I_{\mathrm{MFC}}=1.674$ Nm^2^.

The MFC is a composite of PZT-5A and epoxy. Since it consists of 60% PZT and 40% epoxy by volume, the effective Young’s modulus of the MFC can be calculated using the rule of mixtures: the $E_{\mathrm{PZT}\text{-}5A}$ for PZT-5A is approximately 66 GPa (it varies but is around 60–70 GPa). That of epoxy resin is around 3 GPa.

Thus, $E_{MFC}=0.6\times 66+0.4\times 3=39.6+1.2=40.8$ GPa.

Transformed width of MFC: $W_{MFC}=14\;\mathrm{mm}\times \left(40.8/70\right)=8.4$ mm.

The total EI for the composite section is as follows. The length of the beam (L) = 400 mm = 0.4 m. The MFC is from 0.065 m to 0.15 m, so the length is 0.085 m. The rest is 0.4 − 0.085 = 0.315 m.

To find the effective flexural rigidity (EI) for the entire beam, the average value of EI is

$${\mathrm{EI}}_{\mathrm{avg}}=\frac{{\mathrm{EI}}_{\mathrm{Al}}\times 0.315+{\mathrm{EI}}_{\mathrm{MFC}}\times 0.085}{0.4}=1.4575\;{\mathrm{Nm}}^{2}.$$

The composite beam has aluminum and an MFC, and the MFC is also a combination of PZT and epoxy.

$m_{\mathrm{Al}}=\rho_{\mathrm{Al}}\times A_{\mathrm{Al}}=2700\times 24-6=0.0648\;\mathrm{kg}/m$ and $m_{\mathrm{MFC}}=\rho_{\mathrm{MFC}}\times A_{\mathrm{MFC}}=5160\times 5.32e-6=5160\times 5.32e-6=0.0014\;\mathrm{kg}/m$

Total mass = 0.0648 + 0.0014 = 0.0662 kg

The mass per unit length in the MFC section is (μ): $\frac{\rho_{\mathrm{Al}}A_{\mathrm{Al}}+\rho_{\mathrm{MFC}}}{0.4}=0.1655$ kg/m. Thus, the total mass per unit length varies: 0.162 kg/m for 0–0.065 m and 0.15–0.4 m and 0.1895 kg/m for 0.065–0.15 m.

Applying boundary conditions for a cantilever beam (fixed at x = 0, free at x = L) leads to the characteristic equation for αL, which determines the natural frequencies. The damping ratio ζ used in this analytical model is within the range of the roots of 0.01–0.02.

The characteristic equations $\alpha_{n}$ for the *n*-th mode of a cantilever beam are as follows: α1 = 1.875, $\alpha_{2}$ = 4.694, $\alpha_{3}$ = 7.855, and $\alpha_{4}$ = 10.996 from Equation (54). The first natural frequency is

$$f_{1}=\left(\frac{\alpha_{1}^{2}}{2\pi }\right)\sqrt{\frac{{\mathrm{EI}}_{\mathrm{avg}}}{m_{\mathrm{avg}}L^{4}}}=\frac{{\left(\alpha_{1}L\right)}^{2}}{2\pi }\sqrt{\frac{{\mathrm{EI}}_{\mathrm{composite}}}{\mu L^{4}}}=10.693\;\mathrm{Hz}.$$

**Figure 6:**
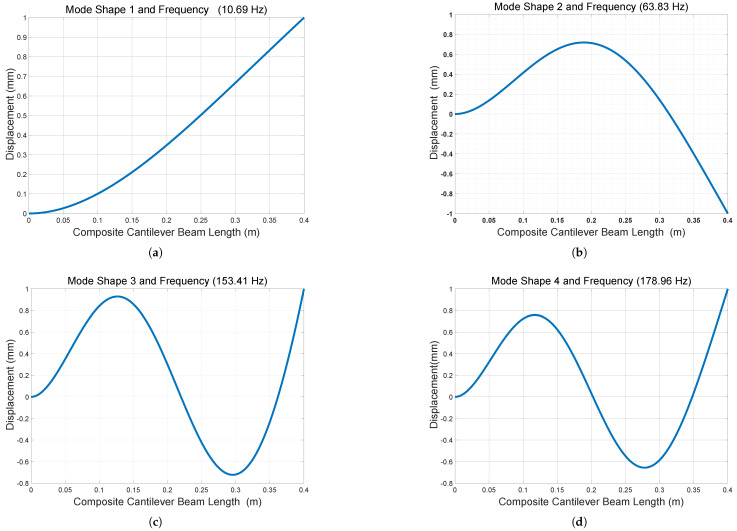
Analytical modeling of eigenfrequency and mode shape by line graph using MATLAB: (**a**) Mode shape and natural frequency one. (**b**) Mode shape and natural frequency two. (**c**) Mode shape and natural frequency three. (**d**) Mode shape and natural frequency four.

The fundamental resonance frequency of 10.69 Hz of the composite beam for the energy harvester can be found in Figure 5 above. The natural frequencies, which are analytically calculated, and the mode shapes, which are modeled by MATLAB R2024b, are shown in Table 2 and Figure 6, respectively. Based on the analysis of the analytical modeling determines the eigenfrequencies and mode shapes of the composite beam. The first eigenfrequency is selected because it is nearly equal to the resonance frequency, which results in the maximum deflection, stress, voltage, and power.

The first eigenmode demonstrates the movement of the energy harvesters in both the positive and negative z-directions, aligning with the resonance frequency of the structure. This deformation shape is optimal for energy harvesting operations. Since these energy harvesters resonate within the low-frequency range, their configurations are particularly effective in capturing low-frequency mechanical vibrations from the surrounding environment.

The maximum deflection for a composite cantilever beam (X) at the free end with an external point load (P) is

$$X=\frac{F\times x^{2}}{6\mathrm{EI}}(3L\;-\;x)=\frac{F}{K\times Q}=3.32\;\mathrm{mm},$$

where a dynamic magnification factor is included. The results of all the analytical models are summarized in the table as shown in Table 3.

### 3.3. Numerical Modeling

**Figure 7:**
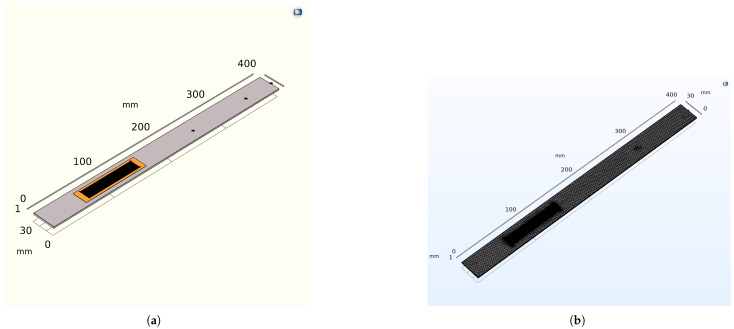
Geometric model and mesh of a cantilever beam with an integrated microfiber composite transducer. (**a**) A geometric model of an aluminum cantilever beam and MFC. (**b**) A geometric, finer mesh of both the substrate and piezoelectric transducer.

The eigenvalues and eigenfunctions of the integrated cantilever beam and a macro fiber composite (MFC) under boundary conditions at the free end, without any external excitation, are determined. The natural frequency (eigenvalues) refers to the frequency at which a system oscillates in the absence of any driving force or damping. The mode shape (eigenfunction) deformation, or displacement of a structure undergoing vibration occurs at its natural frequency. Using an analytical method based on Euler–Bernoulli theory, the natural frequency and mode shape are determined by solving the differential equations provided in Equations (61) and (62), along with the appropriate boundary conditions. COMSOL Multiphysics 6.29 is used for simulation purposes. The cantilever beam is composed of an aluminum base layer, a piezoelectric material (PZT-5A), and epoxy resin. The upper layer has a volume fraction of (0.6%) for PZT-5A and (0.4%) for epoxy resin, with copper used for the electrode component in the numerical simulations. The bottom of the piezoelectric layer is the surface terminal, and the top surface is assigned as a ground condition to the top surface of the piezoelectric layer. An average constant force (0.2–2 N) is applied at the free end of the cantilever beam. The poling direction $d_{31}$ is normal to the plane of the patches. In this configuration, the stress along the poling direction is zero. Regarding the electric field, it is assumed that $E_{3}$ is aligned with the polarization vector, and, along the longitudinal axis, the electric field is zero ($E_{1}$ = $E_{2}=0$). The natural frequencies and corresponding mode shapes for the first four are analyzed numerically using COMSOL Multiphysics simulations. Figure 7 the cantilever beam and the macro fiber composite (MFC) are effectively discretized for finite element analysis using a free tetrahedral mesh with fine element sizes. The natural frequencies are obtained through numerical analysis, demonstrating a favorable dynamic response and excellent compatibility with the vibrating host structure (resonance frequency).

**Figure 8:**
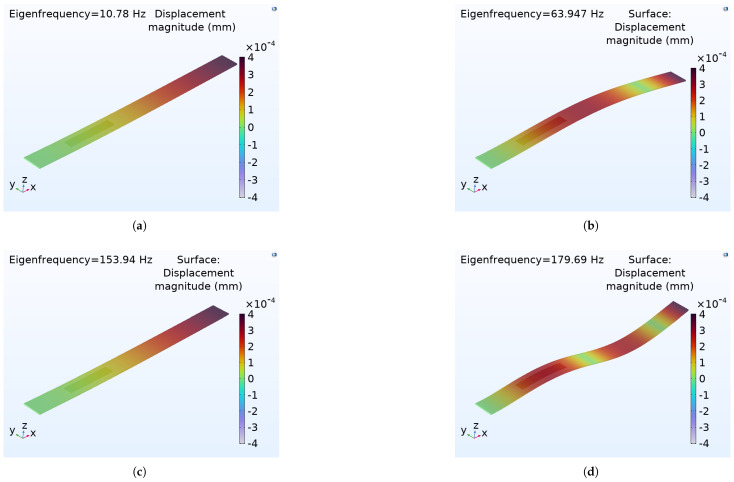
The first four mode shapes and eigenfrequency modeling of an MFC integrated within an aluminium cantilever beam. (**a**) Fundamental bending mode (the first mode is simple bending with the tip moving the most). (**b**) One node is somewhere along the beam (the beam bends upwards, and another part bends downwards). (**c**) Two nodes (three curvature reversals or a twisted shape). (**d**) Three nodes (curvature regions).

**Figure 9:**
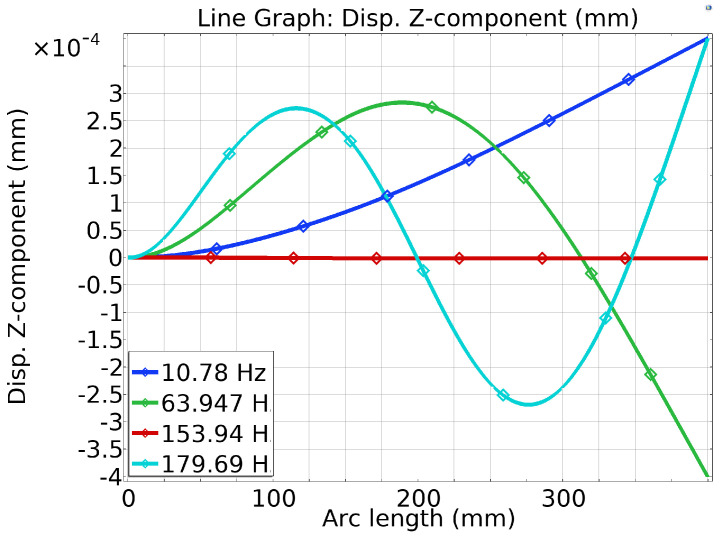
Natural frequency and mode shape shown as line graph.

As the composite beam should be excited at its first resonance frequency, where it experiences the largest deflections, the maximum electrical power comes from piezoelectric elements attached to a vibrating beam at its natural frequency. In this simulation model, the MFC-Al composite beam is excited at the first natural frequency of ≈10.78 Hz. The eigenfrequency for the Al beam without the MFC is ≈10.5 Hz. In Table 4, the natural frequency and angular frequency are modeled by COMSOL Multiphysics, arranged in a tabular form. Figure 8, eigen (natural) frequency and mode shape of geometrical cantilever composite beam (Al-MFC) are simulated. The natural frequency and mode shape for vibration analysis to visualise structural behaviour, and Identify Resonant Conditions by line graph, as shown in Figure 9.

**Figure 10:**
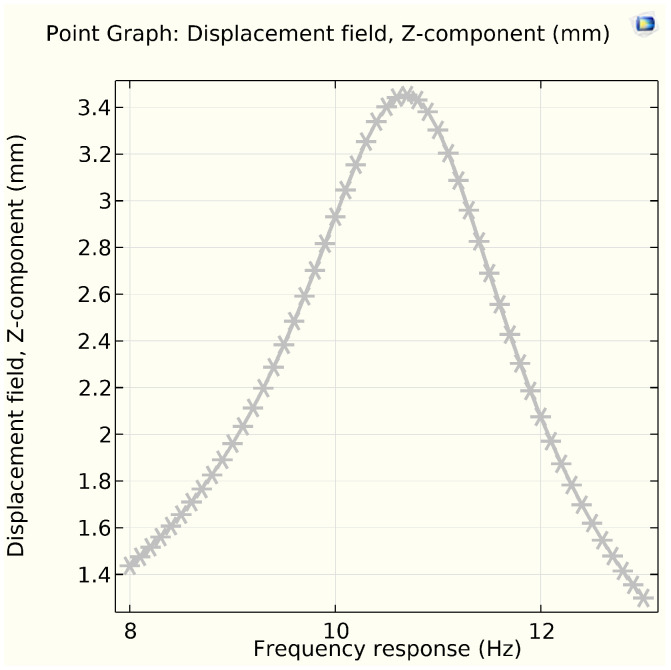
Frequency response vs. displacement (amplitude) in Z-component.

In Figure 10, the frequency versus displacement (amplitude) for the mechanical vibration of the composite aluminum beam within the macro fiber piezoelectric transducer is shown. The results indicate that the optimal displacement occurs at the resonance frequency of the energy harvester, which is 10.78 Hz. Additionally, the data show that the magnitude of displacement increases as the input harmonic force rises from 0.2 N to 2 N over an average duration of 0.1 to 0.5 s.

**Figure 11:**
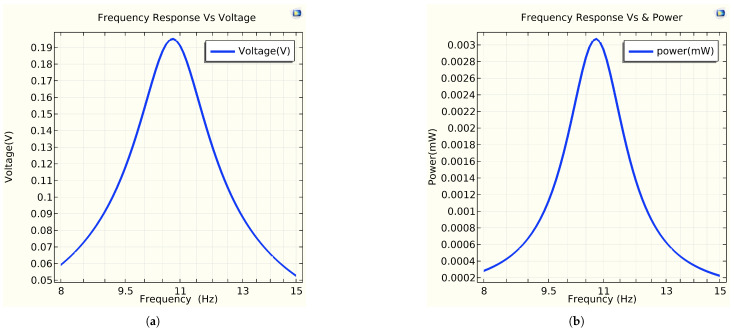
Frequency response vs. voltage and power. (**a**) Frequency response vs. voltage (V). (**b**) Frequency response vs. power (mW).

**Figure 12:**
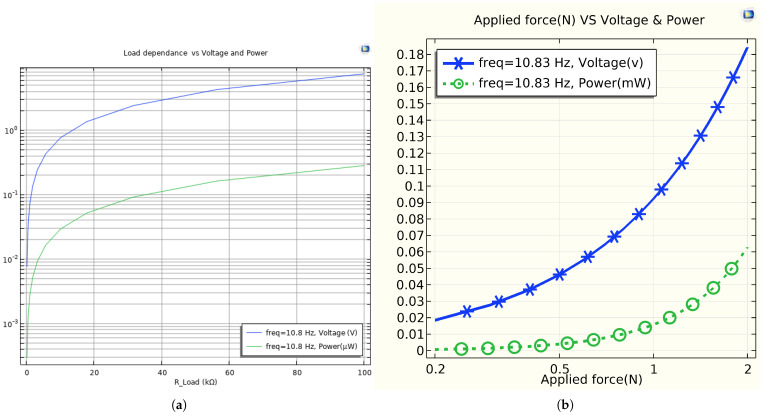
Comparison between the resistance load and the applied external load with voltage and power. (**a**) The power harvested from a macro fiber composite (MFC) as a function of the electrical resistance load at a fixed frequency of 10.78 Hz with a load impedance of 12 kΩ. (**b**) The voltage and electrical power output versus the average dynamic force.

The main objective of a piezoelectric material is to generate a voltage and electric power when a mechanical force is applied. When a force is exerted, charges of opposite polarities (+ and −) are induced in the piezoelectric material. Figure 11a,b illustrate the frequency response in terms of voltage and power, respectively, generated at a resonant frequency of 10.78 Hz. Both the mechanical power harvested in mW and the peak voltage induced across the piezoelectric unimorph in V are measured as a function of the frequency when the energy harvester is excited by an external dynamic force. The voltage and power amplitude decrease as the natural frequency increases. The maximum voltage and power are achieved in the first mode of excitation. Based on Figure 11, the energy harvester must be tuned around the resonance frequency to maximize the electrical power output. In the simulation, the maximum voltage and power were measured to be ≈0.1952 V and 0.0031 mw at 10.78 Hz, respectively. From Figure 12a, the variation in the output voltage and power as a function of the load for the resonance frequencies. Maximum power of 0.0031 mW was measured across the matching load (purely resistive load Z = R) of 12 kΩ at 10.78 Hz with a power density of $0.0169\;{\mathrm{mW}/\mathrm{mm}}^{3}$. From Figure 12b, the resonance frequency, measured at 10.83 Hz, refers to the frequency of a composite beam when subjected to an average force ranging from 0.2 to 2 N, and the natural frequency is simulated with a free load of the beam at 10.78 Hz. This indicates the resonance frequency at which a system responds with maximum amplitude to an externally applied force. The variation between these frequencies is only 0.05 Hz, which represents the interaction between the surface materials used for energy harvesting and the applied force. Generally, the difference between natural frequency and resonance frequency is less than 0.1%. Additionally, the output power for both frequencies remains the same. This illustrates the main importance of the resonance frequency for effective energy harvesting.

### 3.4. Comparison of Analytical and Numerical Power Output

**Figure 13:**
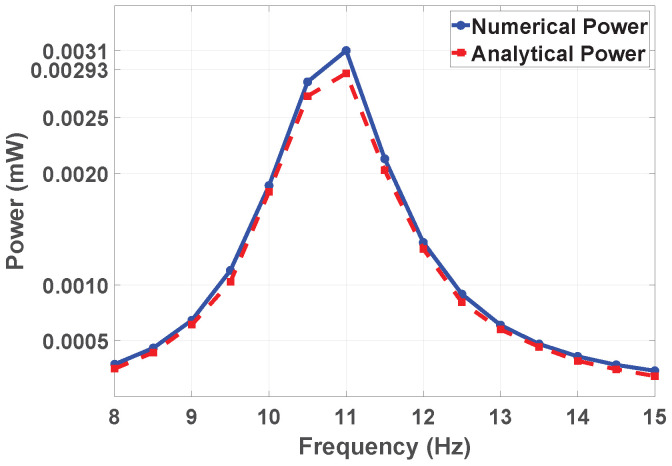
Frequency versus numerical and analytical output power (mW).

As showin in Figure 13, the output power obtained using both analytical and numerical methods across the resonance frequency range of 10.65 and 10.5 Hz is the peak power near the resonance frequency, respectively. For analytical methods, the peak (maximum) power is approximately 0.0031 mW. Meanwhile, for numerical simulations, the maximum electrical output is around 0.0029 mW. The shapes of both graphs are similar. The analytical graph is not as smooth as that of the numerical approach because it reflects the assumptions of linearity and the simplified boundary conditions. Therefore, the analytical method is mostly useful for initial approximation, while the numerical approach is suitable for detailed analysis and performance optimization.

## 4. Conclusions

Piezoelectric energy harvesting via mechanical vibration has garnered greater attention than electrostatic or electromagnetic methods due to its higher power density, scalability, and simpler circuitry. This study integrates a rectangular cross-section MFC-P2 transducer into an aluminum cantilever beam. The model is analyzed using Euler–Bernoulli beam theory and validated through numerical simulations in COMSOL Multiphysics. Owing to its flexibility, durability, and long lifespan, the macro fiber composite (MFC) demonstrates effective energy harvesting from environmental vibrations. For this analysis, rotary inertia and shear deformation effects are neglected under the assumption of a long, slender beam. A key challenge in modeling the $d_{31}$-type macro fiber composite (MFC) piezoelectric transducer lies in the limited availability of mechanical and electrical property data for these structures. This piezoelectric material generates a usable direct voltage for electronic devices while exhibiting minimal mechanical damping. However, a critical limitation of the rectangular geometry arises when a force is applied at the free end: the strain distribution becomes non-uniform, decreasing linearly from the fixed end to the free end, where the strain vanishes entirely. Consequently, the average strain peaks near the fixed end support. Mechanical–electrical coupling effects are incorporated to derive the output power and voltage. The mechanical response of the beam and the electrical output of the MFC transducer are modeled mathematically and simulated numerically. The power generated by a single MFC-based cantilever energy harvester is sufficient to operate ultra-low-power electronic devices, including the Texas Instruments LMT70 temperature sensor in sleep mode, low-power humidity sensors functioning intermittently through sleep cycles, and nano-power real-time clocks (RTCs), such as the Maxim DS3231 in standby mode. For future work, artificial intelligence (AI) could be used to optimize the output voltage and power by identifying the substrate material’s resonance frequency and refining key parameters such as the geometry, material properties, transducer placement, and electrical loading.

## Figures and Tables

**Table 1:** Material properties and dimensional parameters of the vibration energy harvester.

Type of Material	Item	Value	Dimension (Unit)
Aluminum beam (substrate)	Length $\left(L_{Al}\right)$	400	mm
	Width (W)	30	mm
	Thickness $\left(t_{Al}\right)$	2	mm
	Centroid of the aluminum	1	mm
	Area $\left(A_{Al}\right)$	607	mm^2^
	Centroid ($h_{1Al}$)	1	mm
	Poisson ratio	0.345	–
	Elasticity	70	GPa
	$I_{\mathrm{Beam}}$	$2\times 10^{-11}$	m^4^
M8514-P2	Length $\left(L_{\mathrm{MFC}}\right)$	85	mm
	Width $\left(W_{\mathrm{MFC}}\right)$	14	mm
	Thickness $\left(t_{\mathrm{MFC}}\right)$	0.38	mm
	$A_{\mathrm{MFC}}$	5.32	mm^2^
	Centroid of the MFC	2.19	mm
	Total volume	452.2	mm^3^
	$A_{\mathrm{MFC},\;\mathrm{Transformed}}$	3.192	mm^2^
	Centroid MFC ($h_{2}$)	2.19	mm
	$\rho_{\mathrm{MFC}}=0.6\rho_{\mathrm{PZT}}+0.4\rho_{\mathrm{epoxy}}$	5160	kg/m^3^
	Young’s modulus of the M8514-P2	30 × $10^{9}$	Pa
	Elastic compliance constant	$6.4\times 10^{-12}$	${\frac{m}{N}}^{2}$
	Piezoelectric constant coefficient $\left(d_{31}\right)$	$-171\times 10^{-12}$	C/N
	Capacitance of MFC ($C_{p}$)	$45\times 10^{-9}$	F
	$I_{bMFC}$	$6.4017\times 10^{-14}$	m^4^
	Relative permittivity $\epsilon_{r}$	1700	—
Copper electrode	Length	101	mm
	Width	14	mm
	Thickness	0.01	mm
PZT-5A	Length	0.3	mm
	Width	14	mm
	Thickness	0.38	mm
	Number of cells	170	–
	Volume of one cell	1.596	${\mathrm{mm}}^{3}$
	$\rho_{\mathrm{PZT}\text{-}5A}$	7800	${\mathrm{kg}/m}^{3}$
	Volume fraction of PZT	0.6	–
	Total volume of PZT-5A	271.32	mm^3^
	Piezoelectric constant coefficient $\left(d_{31}\right)$	$-171\times 10^{-10}$	m/v
Epoxy resin	Length	0.2	mm
	Width	14	mm
	Thickness	0.38	mm
	Number of cells	170	–
	Volume of one cell	1.064	mm^3^
	Volume fraction	0.4	–
	Total volume of epoxy resin	180.88	mm^3^

**Table 2:** The first six natural frequencies of analytical modeling.

Mode Shape	$\alpha_{n}L$ Roots of the Frequency Equation	Natural Frequency (Hz)	Angular Frequency (rad/s)
1	1.875	10.693	67.578
2	4.694	63.82	397.96
3	7.855	153.41	961.75
4	10.996	178.96	1124.14
5	14.137	269.55	1693.63
6	17.278	355.07	12,218.72

**Table 3:** Summary of analytical results in table form.

Parameter	Formula	Result	Unit
Centroid of Aluminum Beam ($C_{\mathrm{Al}}$)	$C_{\mathrm{Al}}=\frac{h_{\mathrm{Al}}}{2}$	1	mm
Centroid of Al and MFC $\left(C_{Al-MFC}\right)$	$2+\frac{h_{MFC}}{2}$	2.19	mm
Composite Moment of Inertia	$E_{\mathrm{Al}}I_{\mathrm{Al}}+E_{\mathrm{MFC}}I_{\mathrm{MFC}}$	1.674	Nm^2^
Total Mass per Unit Length (μ) of Composite Beam	$\rho_{\mathrm{beam}}A_{\mathrm{beam}}+\rho_{\mathrm{MFC}}A_{\mathrm{MFC}}$	0.1655	kg/m
Total Second Moment $\left(I_{\mathrm{total}}\right)$	−−−	−−	mm
Resonance Frequency $\left(\omega_{n}\right)$	$\frac{{\left(\alpha_{1}L\right)}^{2}}{2\pi }\sqrt{\frac{EI_{\mathrm{composite}}}{\mu L^{4}}}$	10.693	Hz
Maximum Amplitude (*X*)	$\frac{PL^{3}}{3E_{eq}I_{eq}}$	3.3	m
Strain $\left(\epsilon_{x}\right)$	—	—	–
Maximum Stress ($\sigma_{\max}$)	$\frac{M\cdot y}{I_{\mathrm{composite}}}$	1.33	M Pa
Electric Displacement	$d_{31}$ $\times E_{3}\;$ ×ϵ	$0.724\;\times \;10^{-6}$	${C/m}^{2}$
Voltage Output (V)	$\frac{\theta \cdot \omega \cdot X}{{\left(\omega C_{p}\right)}^{2}+{\left(1/R\right)}^{2}}$	0.175	V
Power Output (P)	$\frac{{\left(\frac{d_{31}Y_{p}h_{p}h_{b}}{2\;\varepsilon_{33}^{T}}\int_{0}^{L_{p}}\frac{\partial^{2}W_{1}}{\partial x^{2}}dx\cdot \eta_{\max}\right)}^{2}}{2R_{\mathrm{load}}}$	0.0029	mW

**Table 4:** The first six natural frequencies of numerical modeling.

Mode Shape	Natural Frequency (Hz)	Angular Frequency (rad/s)
1	10.781	67.738
2	63.949	401.81
3	153.94	967.26
4	179.7	1129.1
5	271.15	1703.7
6	355.08	2231.1

## Data Availability

All relevant data are included within the article to support the findings of this study.

## Abbreviations

MFC	Macro fiber composite
COMSOL	Computational Solutions
PZT	Lead zirconate titanate
PEH	Piezoelectric energy harvester
S	Strain
$E_{0}$	Electric field
EM	Electromagnetic
PZ	Piezoelectric
ES	Electrostatic
V	Voltage
IEEE	Institute of Electrical and Electronics Engineers
σ	Stress
D	Electric displacement
$s^{E}$	Compliance matrix at constant electric field
d	Piezoelectric strain coefficient
$h_{p}$	Thickness of the piezoelectric layer
$c_{11}$	Elastic modulus of the piezoelectric layer
$e_{31}$	Piezoelectric stress constant
x	Position along beam length
$\rho_{eq}$	Equivalent density
*A*	Area
E	Elastic modulus
*Q*	Electric charge
α	Damping effect
*P*	Power
PVDF	Polyvinylidene fluoride
*R*	Resistance load
$\omega_{n}$	Angular frequency
$f_{n}$	Natural frequency
